# Mechanically induced polyamorphism in a one-dimensional coordination polymer†

**DOI:** 10.1039/d4sc07058e

**Published:** 2024-11-25

**Authors:** Taichi Nishiguchi, Yuki Ohara, Kentaro Kadota, Xin Zheng, Shin-ichiro Noro, Satoshi Horike

**Affiliations:** a Department of Synthetic Chemistry and Biological Chemistry, Graduate School of Engineering, Kyoto University Katsura, Nishikyo-ku Kyoto 615-8510 Japan; b Department of Chemistry, Graduate School of Science, Kyoto University Kitashirakawa-Oiwakecho, Sakyo-ku Kyoto 606-8502 Japan horike.satoshi.3r@kyoto-u.ac.jp; c Faculty of Environmental Earth Science, Hokkaido University Kita 10, Nishi 5, Kita-ku Sapporo 060-0810 Japan; d Institute for Integrated Cell-Material Sciences, Institute for Advanced Study, Kyoto University Yoshida-Honmachi, Sakyo-ku Kyoto 606-8501 Japan; e Department of Materials Science and Engineering, School of Molecular Science and Engineering, Vidyasirimedhi Institute of Science and Technology Rayong 21210 Thailand

## Abstract

We created different amorphous structures of a coordination polymer by applying mechanical shear forces. One-dimensional Cu(Tf_2_N)_2_(bip)_2_ (1, Tf_2_N^−^ = bis(trifluoromethanesulfonyl)imide, bip = 1,3-bis(1-imidazolyl)propane) melted at 245 °C and underwent a glass transition at −10 °C by a static cooling process. 1 formed another amorphous state with a distinct glass transition point of 70 °C under oscillatory shear stress. The difference of orientation in their structures was studied by X-ray absorption fine structure and small-angle X-ray scattering. The reversible transition between the two amorphous states was observed by dynamic mechanical analyses.

## Abstract 2

We fabricated different glass structures of a one-dimensional coordination polymer with/without the application of mechanical forces during the melt-quenching process. Under the shear, the glass has an oriented, anisotropic structure.

## Graphical abstract

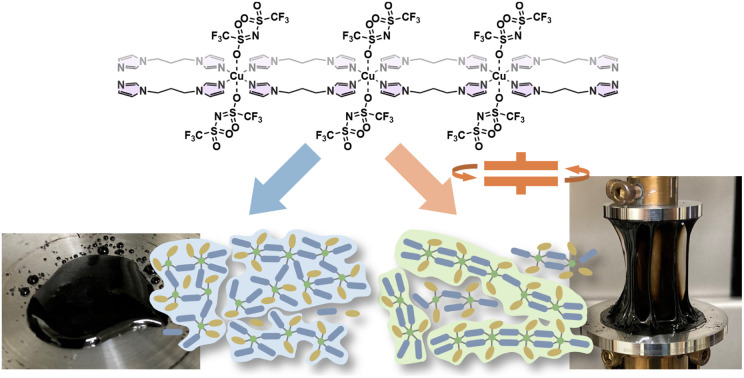

## Introduction

Controlling amorphous structures is one of the most important approaches to designing functional materials. Mechanical processes have been applied to amorphous states to change the arrangement of atoms and molecules which affects their properties.^1^ For example, organic polymers are processed by melt spinning methods, where centrifugal forces are applied, and the polymer chains are aligned to form oriented amorphous states. This orientation affects properties such as glass transition temperature, mechanical strength, and optical transparency.^2–4^ Metallic and chalcogenide glasses also exhibit stress-induced alignment resulting in anisotropic optical and elastic properties.^5,6^ The creation of different amorphous states under mechanical stresses is an essential technique for controlling the properties of materials.

Some classes of coordination polymers (CPs) and metal–organic frameworks (MOFs) composed of metal ions and bridging ligands form liquid and glassy states.^7–9^ The glasses show unique properties including porosity, conductivity, and selective gas permeability.^10–12^ In a recent study, carboxylate-based CP glasses were reported, and CP/MOFs have emerged as novel liquid and glassy materials, along with other related materials such as hybrid organic–inorganic perovskites and metal–organic polyhedra.^13–15^ CP/MOF liquid and glasses are amorphous states featuring coordination bonds, and the structures depend on coordination geometry and bond strength.^16–18^ For example, the liquid states of two-dimensional Zn(H_2_PO_4_)_2_(1,2,4-triazole)_2_ and three-dimensional (3D) Zn(imidazolate)_2_ (ZIF-4) are composed of either discrete metal complexes or isotropic networks.^16,17^ It is known that ZIF-4 shows a phase transition in the liquid state by thermal treatments.^19,20^ It forms a low-density state at 292 °C and a high-density state at 317 °C, and the thermal transition between the two liquid states was observed. These suggest that the liquid and glassy states of CP/MOFs would have a variety of structures and the potential to be controlled by external stimuli. On the other hand, there is no study on controlling liquid and glass structures using mechanical forces and the investigation of structures and properties with and without mechanical forces.

We here report the preparation of two distinct amorphous states of CP by controlled melt quenching under mechanical stimuli. We employed a one-dimensional (1D) Cu^2+^-based crystal structure that melts at 245 °C, due to its structural anisotropy, stability, and reasonable viscosity for the processing.^21,22^ Mechanical shear force in the melt state led to an ordered domain in the amorphous structure, and X-ray absorption fine structure and small-angle X-ray scattering represented structural features in the nanometre range. Furthermore, dynamic mechanical analyses suggested a reversible transition between the two amorphous states in mechanical ways.

## Results and discussion

**Fig. 1:**
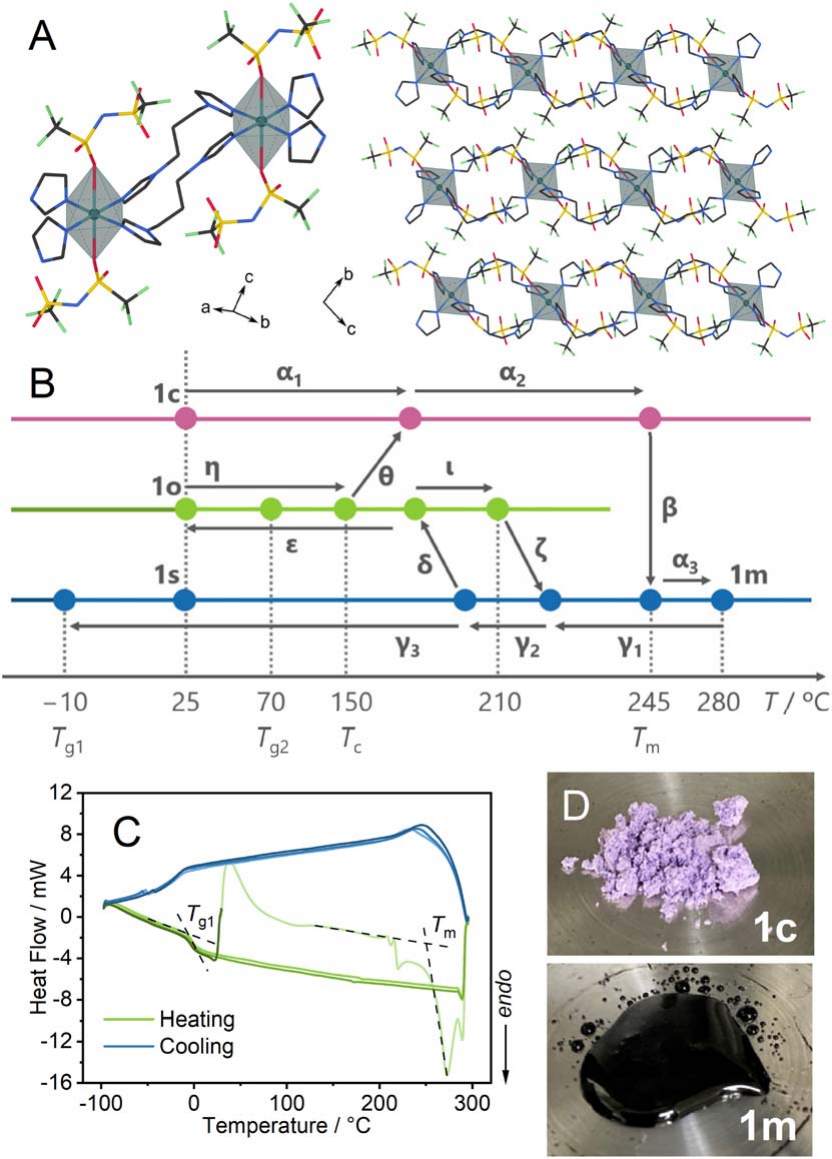
(A) Chain and packing crystal structures of 1c determined at −100 °C. C: grey, N: blue, O: red, F: light green, S: yellow, Cu: dark green octahedra. H is omitted for clarity. (B) Schematic illustration of thermal and mechanical processes and phase-changing behaviours among 1c, 1m, 1s, and 1o. (C) DSC profile of 1c. The melting point (*T*_m_) and glass transition point (*T*_g1_) are indicated by dashed lines. (D) Optical images of 1c (25 °C) and the melt at 280 °C (1m).

Single crystal X-ray diffraction (SC-XRD) analysis of Cu(Tf_2_N)_2_(bip)_2_ (Tf_2_N^−^ = bis(trifluoromethanesulfonyl)imide, bip = 1,3-bis(1-imidazolyl)propane) at −100 °C showed that Cu^2+^ has a distorted octahedral coordination to four bip and two Tf_2_N^−^. The bip bridges the Cu^2+^ to construct 1D chain structures (Fig. 1A). The synchrotron powder X-ray diffraction (PXRD) pattern of a bulk powder sample, denoted as 1c, was collected at room temperature under air (Fig. S1†). It matched the simulated pattern from the SC-XRD crystal structure.

### Thermal properties of 1c (α_1_ → α_2_ → β → α_3_ in Fig. 1B)

Thermogravimetric analysis (TGA) of 1c showed a weight loss of 1.4% at 350 °C (Fig. S2†), followed by further weight loss in the higher temperature region corresponding to the thermal decomposition. Differential thermal analysis (DTA) showed an endothermic peak at 245 °C, indicating melting of the crystal. In Fig. 1B, we summarise the thermal and mechanical processes we performed (α–ι), and hereafter we denote each step by Greek letters.

In the heating process of 1c (α_1_ → α_2_ → α_3_) in differential scanning calorimetry (DSC, Fig. 1C), melting (β) was observed at 245 °C (*T*_m_). Variable temperature (VT) PXRD and *in situ* optical monitoring (Fig. 1D) supported that the endotherm in DSC corresponds to the melting. VT-Fourier transformed infrared (FT-IR) spectra at 30–300 °C (Fig. S3 and S4†) showed identical peaks before and after the melting, suggesting the preservation of chemical structures of the components. The peaks at 1324 and 1344 cm^−1^, and the one at 1129 cm^−1^ are assigned to asymmetric and symmetric S=O stretching modes, respectively. The peaks at 1190 and 1060 cm^−1^ evidence the preservation of the vibration of CF_3_ and S–N–S groups.^21^

### Preparation of **1s** under static conditions (γ_1_ → γ_2_ → γ_3_) and characterisation

We heated 1c to 280 °C to obtain a melt, which we refer to as 1m. 1m was cooled down statically to room temperature (γ_1_ → γ_2_ → γ_3_) to prepare 1s (s means static). In the DSC profile of 1s, a glass transition (*T*_g1_) was observed at −10 °C. *T*_g1_ is below room temperature, and 1s is regarded as a supercooled liquid at room temperature. The identical DSC profiles of the two cycles of heating/cooling proved the reversibility of the glass transition behaviour. The ratio of the *T*_g1_/*T*_m_ (K/K) = 0.51 is small compared with the much-reported melting and glass-forming CP/MOFs, suggesting the existence of two different amorphous states corresponding to the respective transition.^9^

**Fig. 2:**
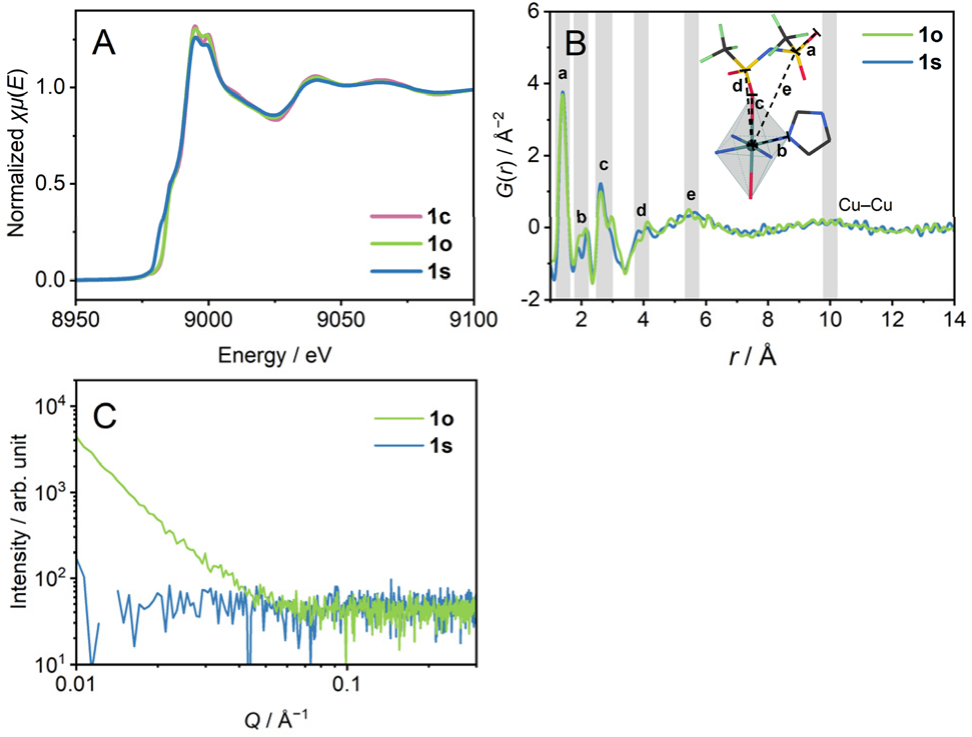
(A) XANES profiles. (B) PDF analysis. *G*(*r*) is plotted to radius. Inset schematic representation indicates the dominantly corresponding distance correlations. (C) SAXS profiles. CuKα X-ray source was used.

X-ray absorption near edge structure (XANES) spectra of the Cu K-edge (Fig. 2A and S5†) confirm the oxidation number of 2+ for 1c and 1s by comparison with Cu, Cu_2_O, and CuO. The XANES spectrum of 1s showed a pre-edge suggesting a lower-symmetric coordination sphere or square-planer coordination.^23^ Extended X-ray absorption fine structure (EXAFS) reveals the preservation of hexa-coordination in 1c and 1s (Fig. S6†). We performed a pair distribution function (PDF) analysis of 1s from X-ray total scattering data (Fig. 2B). The peak at the radius (*r*) of 5.5 Å reflects dominantly the correlation between Cu^2+^ and S atoms. The broad peak at *r* = 10 Å reflects inter- and intra-chain Cu–Cu distance correlations. The distances of intra-chain and inter-chain Cu–Cu are 9.8 Å and 10.4 Å in the crystal structure. Preservation of the Cu–Cu distance correlation suggests the linker-bridged extended structures of 1s.^24^ We conducted small-angle X-ray scattering (SAXS) for a scattering vector (*Q*) of 0.007–0.36 Å^−1^ at room temperature (Fig. 2C). The absence of the intensity increase in the pattern of 1s suggests an isotropically randomised structure without the formation of inhomogeneous fractal domains.^25^

### Preparation of 1o under oscillatory strain conditions (γ_1_ → γ_2_ → δ → ε) and characterisation

1m was cooled down to room temperature at −1 °C min^−1^ under a 0.05% oscillatory strain at the frequency of 10 rad s^−1^ with N_2_ flow (γ_1_ → γ_2_ →δ → ε) to have 1o (o denotes oscillatory strain). We carried out XANES, PDF, and SAXS analyses to investigate the structure of 1o. The XANES spectrum confirms the oxidation number of 2+ (Fig. 2A). PDF of 1o showed an identical curve to that of 1s in the region of *r* < 14 Å, preserving the Cu–Cu distance correlation (Fig. 2B). This indicates the identical middle-range ordered structures in 1s and 1o. On the other hand, SAXS profiles reveal the structural difference between 1s and 1o (Fig. 2C). 1o exhibited an increase in the profile below *Q* of 0.05 Å^−1^. The double logarithmic plot finds a linear trend of scattering intensity of 1o against *Q*, following the power law.^26^ The slope, or the exponent, generally takes the value from 3.0 to 4.0, reflecting the fractal dimension of domains ranging from three to two. The exponent was calculated as *α* = 3.0 for 1o, indicating the hierarchical structure with the fractal dimension of 3.0.^27^*α* = 4 was reported for the Zn_4_O(1,4-benzenedicarboxylate)_3_ (MOF-5) crystal, and *α* = 3.5–3.9 for crystalline zeolitic imidazolate frameworks (ZIFs).^26,28^ For porous crystalline systems, the fractal dimension reflects the internal pore structure, and it decreases upon amorphisation. In contrast to them, 1o possesses a domain structure with a high fractal dimension even in an amorphous state, suggesting hierarchical aggregation of nanometre-scale polymeric structures. The difference of 1s and 1o in the SAXS profiles imitates the amorphisation of covalent organic polymers. For example, the sequential melting of crystalline poly(ethylene naphthalene 2,6-dicarbonate) decreases the scattering intensity to give a monotonic pattern.^25,29^ N_2_ and CO_2_ adsorption and desorption isotherms at 77 K and 195 K observed negligible uptake both in 1s and 1o (Fig. S7†), suggesting the non-porous structures of the samples. The structural difference between 1s and 1o without forming a porous structure supports the dense domain formation in 1o.

We conducted helium pycnometry at 25.0 °C to investigate the densities of 1s and 1o. The densities were 1.711(3) g cm^−3^ for 1s, and 1.729(3) g cm^−3^ for 1o. 1o is 1% denser than 1s, and 1o is a high-density amorphous state, and 1s is a low-density amorphous state. This suggests that the mechanical stimuli influence the packing of the components and the formation of a distinct amorphous state. 1s and 1o are 2 or 3% denser than 1c (1.678 g cm^−3^, as calculated from the SC-XRD structure), indicating a better-packed structure in 1s and 1o. Similar amorphisation-induced densifications were reported in some melting CP/MOFs.^24^

**Fig. 3:**
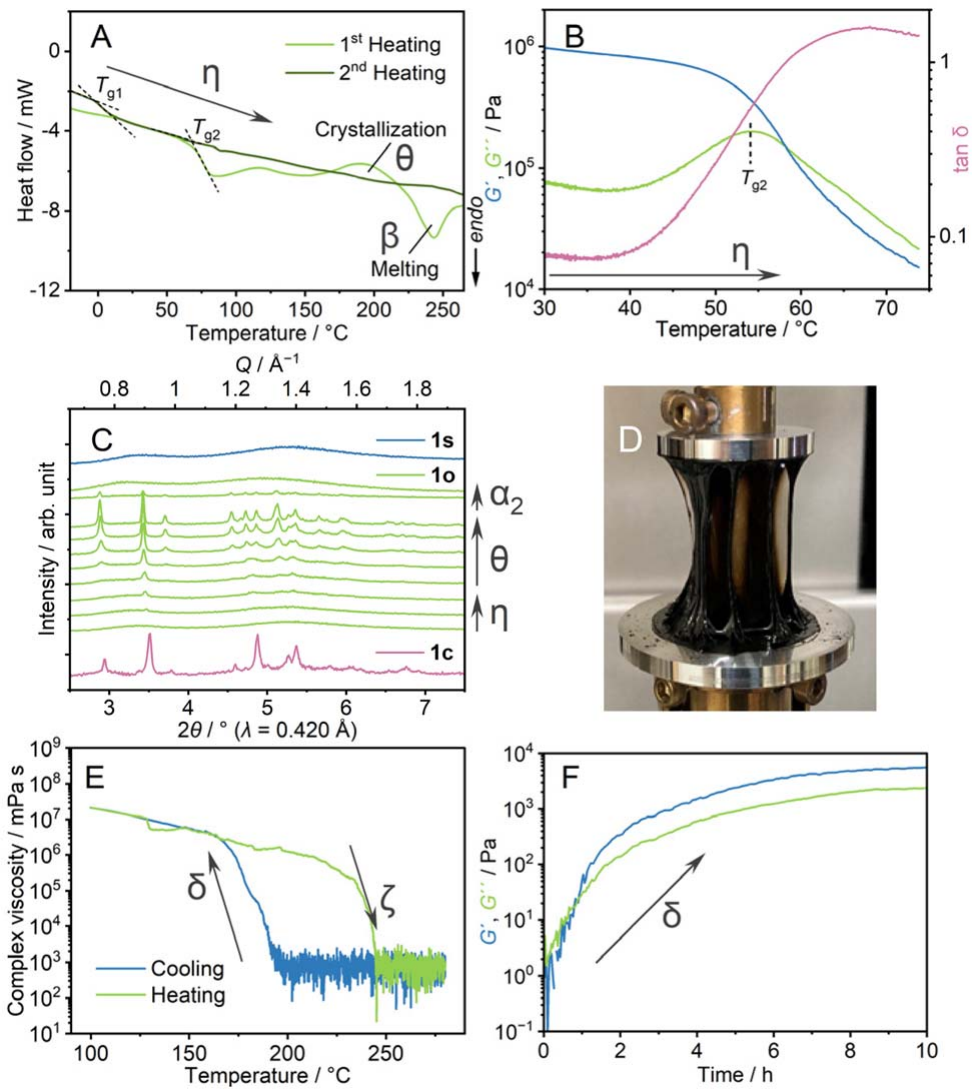
(A) DSC profiles of 1o from −20 to 270 °C under an Ar atmosphere. Glass transition points (*T*_g1_, *T*_g2_) are indicated with dashed lines. (B) Temperature-ramp DMA profile of 1o from 30 to 75 °C. (C) Synchrotron PXRD of 1c (pink), 1s (blue), 1o (pale green). 1o was heated from 30 (bottom) to 270 °C (top). (D) Optical image of 1o heated at 100 °C. (E) The complex viscosity in the temperature range of 100–280 °C. (F) Time-sweep DMA profile at 200 °C.

Thermal behaviours of 1o were characterised by TGA, DSC, and dynamic mechanical analysis (DMA). The TGA profile under an Ar atmosphere showed weight loss at 350 °C (Fig. S8†). This decomposition behaviour is identical to that of 1c. In the heating process of DSC (Fig. 3A, η), 1o showed a glass transition at 70 °C (*T*_g2_), followed by crystallisation (*θ*) at *T*_c_ and melting (α_2_ → β). The ratio of the glass transition and melting points *T*_g2_/*T*_m_ = 0.67 follows Kauzmann's 2/3 law.^30^ This suggests that transitions at *T*_g2_ and *T*_m_ correspond to forming an amorphous structure which is identical to 1o at room temperature. DMA determined a glass transition point as a relaxation mode change at the comparable temperature of 55 °C (Fig. 3B, η). The formation of the same state as 1c above the glass transition is confirmed by VT-PXRD (Fig. 3C). As the *T*_g2_ in DSC is 70 °C, 1o is in a glassy state at room temperature. A scanning electron microscopy (SEM) image (Fig. S9†) showed the formation of a crack-free surface, supporting complete amorphisation from 1c to 1o. 1o gave a viscous thread-forming liquid state upon heating at 100 °C (Fig. 3D).

We applied oscillatory stress with different shear rates and strain under the same cooling rate to study the formation of different amorphous states. We prepared four samples and DSC measurements were conducted (Fig. S10†). The samples prepared under harsh conditions (5% and 10 rad s^−1^, 0.05% and 100 rad s^−1^) showed a glass transition at 30 °C, which is in between *T*_g1_ and *T*_g2_. The *T*_g_/*T*_m_ ratio is 0.59, and possibly the collapse of the structure domain occurs. Upon applied stress with a smaller shear rate (0.05% and 1 rad s^−1^), the sample exhibited an unclear glass transition, suggesting the incomplete formation of a homogeneous state. From these results, the stress with 0.05% strain and 10 rad s^−1^ was suitable to induce the distinct amorphous structure (1o).

### Transformation between 1s and 1o (δ ↔ ζ)

We conducted DMA to investigate the transition behaviour between 1s and 1o (Fig. 3E and F). 1m was cooled down from 280 to 100 °C at the ramping rate of −1 °C min^−1^ (Fig. 3E). The complex viscosities are 8 × 10^2^ mPa s at 190 °C and 8 × 10^6^ mPa s at 170 °C in the cooling process (γ_1_ → γ_2_ → δ → ε). The following heating process from 100 to 280 °C showed a decrease of storage shear modulus (*G*′) and loss shear modulus (*G*′′) at 230 °C (ι → ζ). Four heating/cooling cycles in the measurements showed the hysteric curves of *G*′ and *G*′′ in the identical temperature range, confirming the reversibility of the rheological transition (Fig. S11†).

Isothermal DMA (Fig. 3F) confirms the viscoelastic change at a constant temperature. 1m was cooled down statically to 200 °C at −1 °C min^−1^, and time-sweep DMA was performed at 200 °C. The profile showed evolutions of *G*′ and *G*′′. Upon the increase of *G*′ and *G*′′, the curves crossed at 48 minutes under isothermal conditions, suggesting a structural transformation from a state like 1s and a state that leads to the formation of 1o observed at room temperature. A similar rheological transition was found in molecular liquids. As an example, triphenyl phosphite exhibits a development of *G*′ in the spinodal decomposition process.^31^ In the cooling process of DMA of 1m (Fig. 3E), 1s changed into 1o by applying the oscillatory strain. This explains the different transition behaviours between 1s and 1o. The second heating process of the DSC of 1o (Fig. 3A, η) showed another baseline shift at −2 °C, comparable to *T*_g1_. This suggests the transition from the structure which gives 1o at room temperature, to that forming 1s at room temperature in the melt-cooling processes of DSC of 1o. Two glass transition points, *T*_g1_ and *T*_g2_ for 1s and 1o, are observed in DSC, and the crystallisation behaviour only found in the heating process of 1o supports the different structures of 1s and 1o. Similar 1D and 3D amorphous states were found in inorganic liquids. As a reference, zinc(ii) chloride exhibits a fragile–strong transition between corner- and edge-sharing configurations in the liquid states.^32,33^ The two states are viewed as random 3D networks and 1D chains, and these two liquid states present structural similarities with 1s and 1o.

### Proposed structures and free energy diagram

**Fig. 4:**
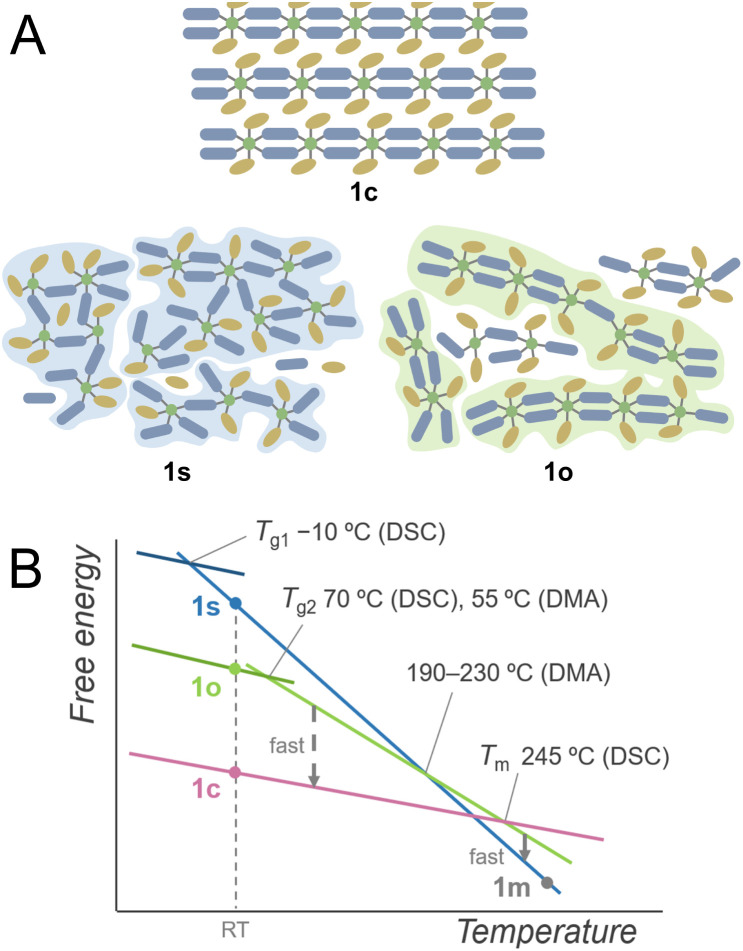
(A) Proposed schematic structures of 1c, 1s, and 1o. Dark blue and ochre indicate bip ligands and Tf_2_N^−^ anions. (B) Proposed schematic free energy diagram.

Based on these investigations, the plausible schematic structures of 1s and 1o are proposed in Fig. 4A. 1s is in an isotropic networked structure, and 1o is in an anisotropic chain-forming structure. Both 1s and 1o form extended structures constructed by Cu^2+^ and linkers as revealed by XANES spectra and PDF analysis. The structural differences appear in the nanometre scale, and the hierarchically assembled structures of 1o are suggested in the SAXS profile. The 1D structures in 1o and 1c suggest a low crystallisation activation barrier in the heating of 1o, which explains the crystallisation observed in the heating of 1o. When 1c is heated, it forms an isotropically randomised liquid with a similar structure to 1s upon melting at 245 °C. A plausible free energy diagram is proposed in Fig. 4B. In the heating process of 1c, it forms a superheated crystalline state without transformation into isotropically random liquid and melts at 245 °C.^34^ The transition from the crystal to isotropic liquid in the superheated region is suppressed due to the high activation barrier to deform the ordered structure into a randomly coordinated state. This is consistent with the too-low *T*_g1_ for *T*_m_ based on Kauzmann's law, suggesting the existence of another melting point corresponding to the same amorphous states corresponding to *T*_g1_. The obtained melt is first in a chain-dissociated state and readily changes into the isotropic state of 1m. In static cooling, 1m gives 1s at room temperature and undergoes a glass transition at −10 °C. In contrast, in cooling 1m under an oscillatory strain, the melt overcomes the barrier from a kinetically trapped isotropic network to stable anisotropic chains, forming 1o at room temperature. When 1o is heated without mechanical stimuli, DSC observes a glass transition at 70 °C, followed by transformation into the original crystalline structure. This crystal melts upon further heating to give a liquid state likewise to 1c. Structural transitions in liquids, including the liquid–liquid transitions and fragile–strong transitions, are evidenced by calorimetric analyses under static conditions such as for silicon, triphenyl phosphite, and ZIFs.^20,35,36^ On the other hand, the DSC of 1c showed no signal assigned to a transition between two liquid states. This suggests that the transition of two amorphous states was induced by applying the oscillatory strain in the thermal processes. This phenomenon originates from the existence of closely stable amorphous states, and the anisotropy and fluidity of the 1D structure also contribute to the formation of different amorphous structures by mechanical forces.

A small *T*_g1_/*T*_m_ ratio was a clue in finding the polyamorphism in 1s and 1o. Relatively high *T*_g_/*T*_m_ ratios were found in polyamorphic liquids, such as triphenylphosphite (0.76), and an ionic liquid of trihexyl(tetradecyl)phosphonium borohydride (0.74).^31,37^ This is due to the liquid phase with higher *T*_g_. In the case of CP/MOFs having diverse structures in the crystalline and liquid phases, they potentially form superheated crystalline states.^38^ In this respect, the experimentally observed *T*_m_ can be higher, making *T*_g1_/*T*_m_ smaller. Although the superheated kinetics and polymorphism in the crystalline phases also influence the *T*_g_/*T*_m_ ratio, anomalously large or small *T*_g_/*T*_m_ indicates the existence of polyamorphism in CP/MOFs.

## Conclusions

In conclusion, we investigated the formation of two distinct amorphous states of 1D crystalline Cu(Tf_2_N)_2_(bip)_2_ by controlling the mechanical shear forces in the melt-quenching process. Two amorphous samples were prepared by melt quenching with and without oscillatory strain. These glasses have different glass transition points and phase change behaviours. DMA showed a viscoelastic jump in cooling the melt under oscillatory strain. SAXS revealed the nanometre-scale structural difference between the two glasses. The results suggest a structural transition upon mechanical stimuli in the liquid state. The technique of mechanically inducing melt-quenching in the liquid state opens a methodology to explore polyamorphism in coordination polymers and MOFs, allowing the discovery of new functional glassy states that have been hidden in conventional vitrification techniques.

## Data availability

The data supporting this article have been included in the ESI.† Crystallographic data for Cu(Tf_2_N)_2_(bip)_2_ have been deposited at the CCDC under 2389504 and can be obtained from https://www.ccdc.cam.ac.uk/structures/.

## Author contributions

T. N. and S. H. conceptualised the project. T. N., Y. O., K. K., Z. X. and S.-I. N. contributed to data collection and formal analyses. T. N. and S. H. wrote the manuscript and all the authors approved the final version.

## Conflicts of interest

There are no conflicts to declare.

## Supplementary Material

SC-OLF-D4SC07058E-s001

SC-OLF-D4SC07058E-s002
