# Supplementary material for: Mechanically induced polyamorphism in a one-dimensional coordination polymer
Source: Chem Sci. 2024 Nov 25;16(2):621–6. doi: 10.1039/d4sc07058e (PMC11616725; doi:10.1039/d4sc07058e)
Supplement: SC-016-D4SC07058E-s001 [file SC-016-D4SC07058E-s001.pdf]

Supplementary Information (SI) for Chemical Science.
This journal is © The Royal Society of Chemistry 2024

Supporting Information for

Mechanically-induced Polyamorphism in One-dimensional Coordination Polymer

Taichi Nishiguchi,[a] Yuki Ohara,[a] Kentaro Kadota,[b] Xin Zheng,[c] Shin-ichiro Noro,[c] Satoshi Horike*[bde]

[a] Department of Synthetic Chemistry and Biological Chemistry, Graduate School of Engineering, Kyoto University, Katsura, Nishikyo-ku, Kyoto 615-8510, Japan
[b] Department of Chemistry, Graduate School of Science, Kyoto University, Kitashirakawa-Oiwakecho, Sakyo-ku, Kyoto 606-8502, Japan
[c] Faculty of Environmental Earth Science, Hokkaido University, Sapporo 060-0810, Japan
[d] Institute for Integrated Cell-Material Sciences, Institute for Advanced Study, Kyoto University, Yoshida-Honmachi, Sakyo-ku, Kyoto 606-8501, Japan
[e] Department of Materials Science and Engineering, School of Molecular Science and Engineering, Vidyasirimedhi Institute of Science and Technology, Rayong 21210, Thailand

Materials and synthesis of crystalline $Cu(Tf_2N)_2(bip)_2$

Bis(trifluoromethanesulfonyl)imide ($H(Tf_2N)$) was purchased from Tokyo Chemical Industry Co., Ltd. Copper (II) hydroxide ($Cu(OH)_2$) was purchased from Fujifilm Wako Pure Chemical Co., Ltd. 1,3-(1-imidazolyl)propane (bip) was purchased from AmBeed Inc. All chemicals and solvents used in the synthesis were of reagent grade and used without further purification.

Crystalline powders of $Cu(Tf_2N)_2(bip)_2$ were synthesized as follows: 20 mmol of $H(Tf_2N)$ was dissolved in 100 mL of H_2O, and 10 mmol $Cu(OH)_2$ were added to the solution and stirred at room temperature for 1 h. The solvent was evaporated, and the precipitate was dried at 80 °C for 6 h under vacuum. The obtained blue crystals were dissolved in H_2O, and a 50 mL of 0.10 M $Cu(Tf_2N)_2$ aqueous solution was prepared. The solution was added dropwise to a 50 mL 0.2 M bip MeOH solution and stirred at room temperature for 2 h. The purple precipitates were collected, washed with MeOH, and dried at 80 °C for 6 h under vacuum. Single crystal of $Cu(Tf_2N)_2(bip)_2$ was synthesized in three-layered solutions of 0.10 M $Cu(Tf_2N)_2/H_2O$, H_2O/MeOH 1/1 mixture, and 0.20 M bip/MeOH.

Single crystal X-ray diffraction and structure (SCXRD) analysis

SCXRD measurements were performed with a Rigaku Micro Max-007 HF diffractometer, a Pilatus 200 K detector, and CuKα radiation (λ = 1.54184 Å) at 173 K. The crystal structure was solved by the direct method SHELXT[1] and refined by the use of full-matrix least-squares techniques on F2 in SHELXL-2018 (ver. 2018/3).[2] The crystal structure of $Cu(Tf_2N)_2(bip)_2$ was deposited in the Cambridge Crystallographic Data Center (CCDC) with the number 2389504.

Powder X-ray diffraction (PXRD)

The samples were sealed in glass capillaries with a diameter of 0.2 mm. The PXRD data were collected using synchrotron radiation (λ = 0.420 Å), employing a large Debye-Scherrer camera and semiconductor detectors on the BL02B2 beamline at the Super Photon Ring (SPring-8, Hyogo, Japan).

Thermal property measurements

Thermogravimetric and differential thermal analyses (TG-DTA) were performed with a Rigaku Thermo plus TG 8120 for the temperature range between 30 and 500 °C under 50 mL min^{-1} of argon gas flow. The heating rate was 10 °C min^{-1}. Differential scanning calorimetry (DSC) was conducted with a Hitachi DSC7200. The samples for DSC were sealed in aluminum pans under an argon atmosphere.

Fourier-transformed infrared (FT-IR) spectroscopy

FT-IR spectra were collected with a Nicolet iS5 equipped with GladiATR (PIKE Technologies Inc.) accessory under a nitrogen atmosphere. The measurements were performed from 25 to 300 °C for the wavenumber of 4000 to 500 cm^{-1}. The background was recorded and subtracted from the collected sample data at each temperature.

Dynamic mechanical analyses (DMA)

DMA was performed with TA Instruments Discovery Hybrid Rheometer HR20. A pair of 25 mm diameter parallel plates were used. An oscillatory strain of 0.05% at the frequency of 10 rad s^{-1} was applied under a flowing nitrogen atmosphere.

X-ray absorption spectroscopy (XAS)

The samples were mixed with boron nitride and pressed into pellets with a diameter of 10 mm and a thickness of 1.0 mm. The synchrotron X-ray absorption spectra for the energy region of the Cu *K*-edge were collected in transmission mode at the BL14B2 beamline in the Super Photon ring-8 GeV (SPring-8, Hyogo, Japan). Fourier

transformation was k^2-weighted in the k range of 3.0 to 12.3 $Å^{-1}$. The data processing and fitting were performed with Athena and Arthemis software.

X-ray scattering and pair distribution function (PDF) analyses

The samples were sealed in quartz glass capillaries with a diameter of 2.0 mm under an argon atmosphere. The synchrotron X-ray total scattering patterns were collected at the BL04B2 beamline in the Super Photon ring-8 GeV (SPring-8, Hyogo, Japan) by four CdTe and two Ge detectors for the scattering vector Q range up to 25 $Å^{-1}$ The energy of the beam was 61.377 keV (λ = 0.2020 Å). The scattering data was applied absorption, background, and Compton scattering corrections, and normalized to give the Faber–Ziman total structure factor $S(Q)$. The pair distribution functions (PDFs) were calculated by Fourier transforming the $S(Q)$ with a Lorch modification function.[3–5]

Small-angle X-ray scattering (SAXS)

SAXS profiles were measured with Rigaku SmartLab multipurpose diffractometer with CuKα radiation (λ = 1.54 Å) equipped with a SAXS attachment. The samples were filled in a quartz glass capillary with a diameter of 2.0 mm, and measurement was performed at 25 °C.

Scanning electron microscope (SEM)

SEM image was collected with JEOL field-emission scanning electron microscope (JSM-75FCT). The acceleration voltage was set 10 kV.

Gas adsorption measurements

Gas adsorption and desorption isotherms were measured with Microtrac BELSORP-mini at 77 K for N_2, and 195 K for CO_2.

Helium pycnometry

Helium pycnometry was conducted with Microtrac BELPYCNO at 25.0 °C. The data was collected over 30 times with a statistical accuracy of 0.175%.

.

Table S1. Parameters of crystal structures of $Cu(Tf_2N)_2(bip)_2$.

**Formula**	$C_{22}H_{24}Cu_1F_{12}N_{10}O_8S_4$
**Formula weight /g mol^{-1}**	976.29
***T* / K**	173
**Wavelength / Å**	1.54187
**Crystal system**	Triclinic
**Space group**	*P*–1
***a* / Å**	10.3863(2)
***b* / Å**	13.1630(2)
***c* / Å**	14.3802(4)
***α* / °**	90.6959(18)
***β* / °**	101.511(2)
***γ* / °**	104.7863(18)
***V* / Å^3**	1858.52(7)
**Z**	1.90
***ρ* / g cm^{-3}**	1.678
***μ* / mm^{-1}**	3.971
**Number of collected/independent reflection**	7137/6654
**R_1, wR_2 [I□2σ(I)]**	0.0577, 0.1636
**R_1, wR_2 (all data)**	0.0595, 0.1617
**GOF**	1.055

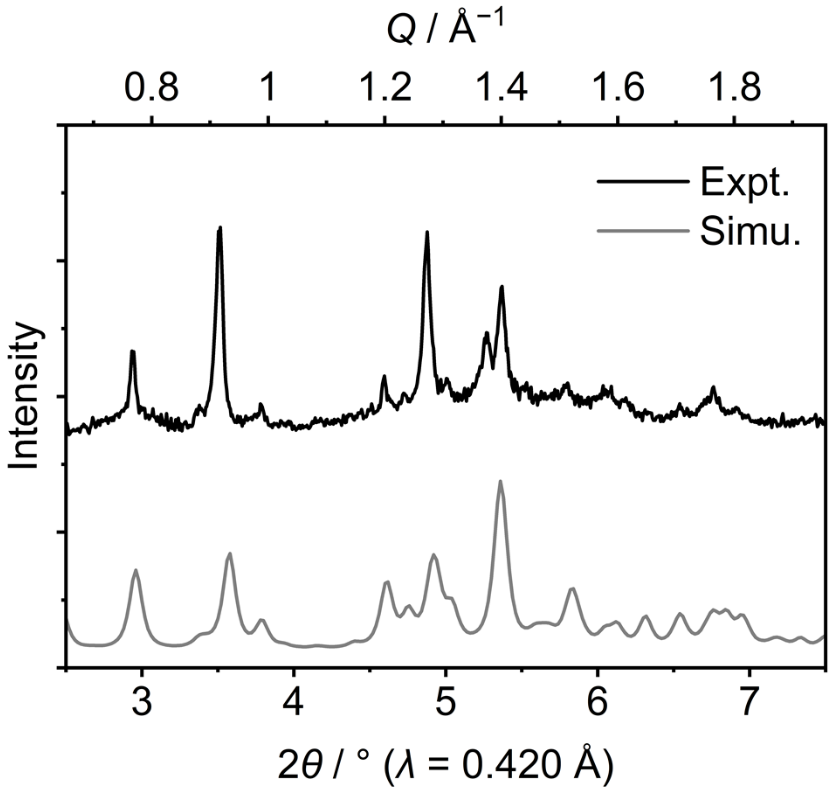

Figure S1. Experimental and simulated PXRD patterns of **1c**. The experimental pattern was collected at 25 °C, and the simulated pattern was obtained from SCXRD data at –100 °C.

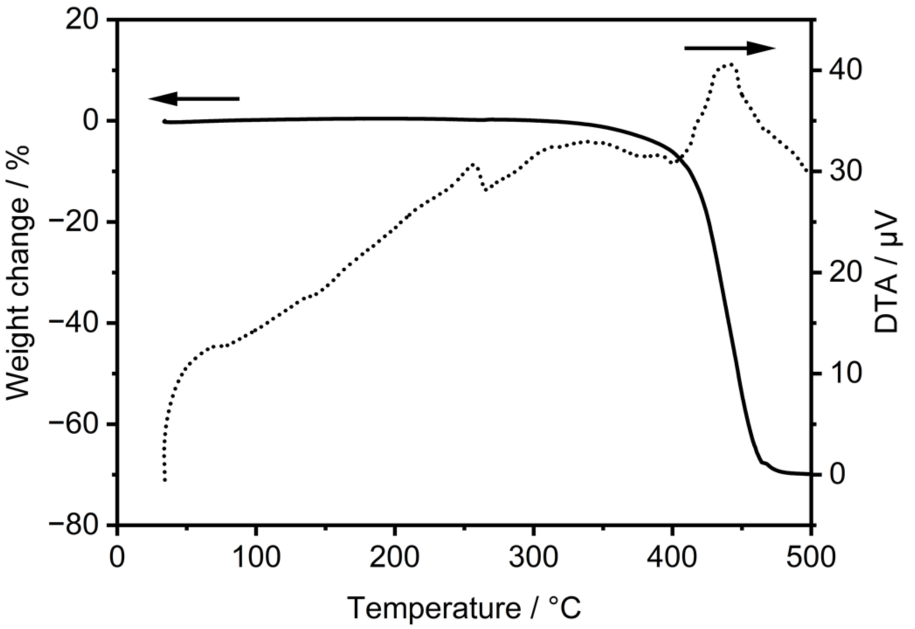

Figure S2. TG-DTA profiles of **1c** from 30 to 500 °C. Solid and dotted lines represent TGA and DTA, respectively.

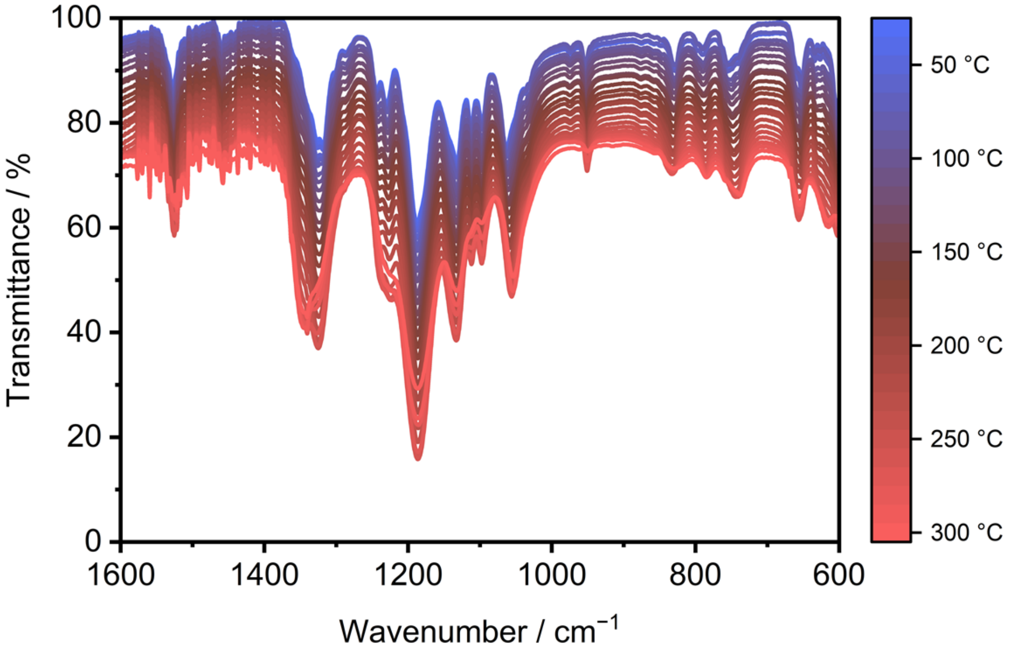

Figure S3. VT-IR spectra of **1c** from 30 to 300 °C.

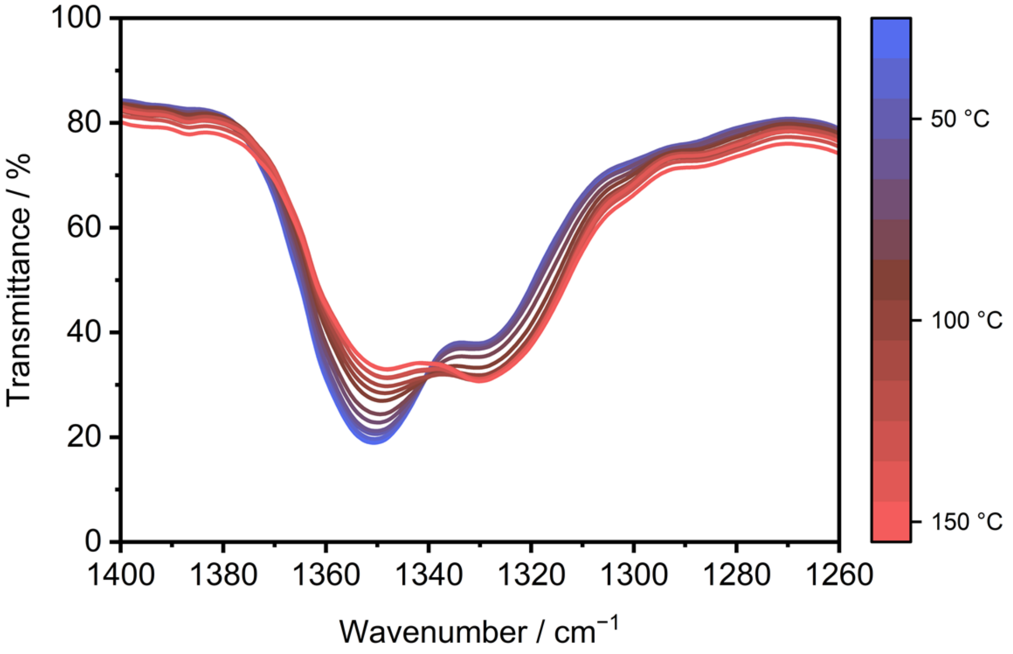

Figure S4. VT-IR spectra of **1c** from 30 to 150 °C.

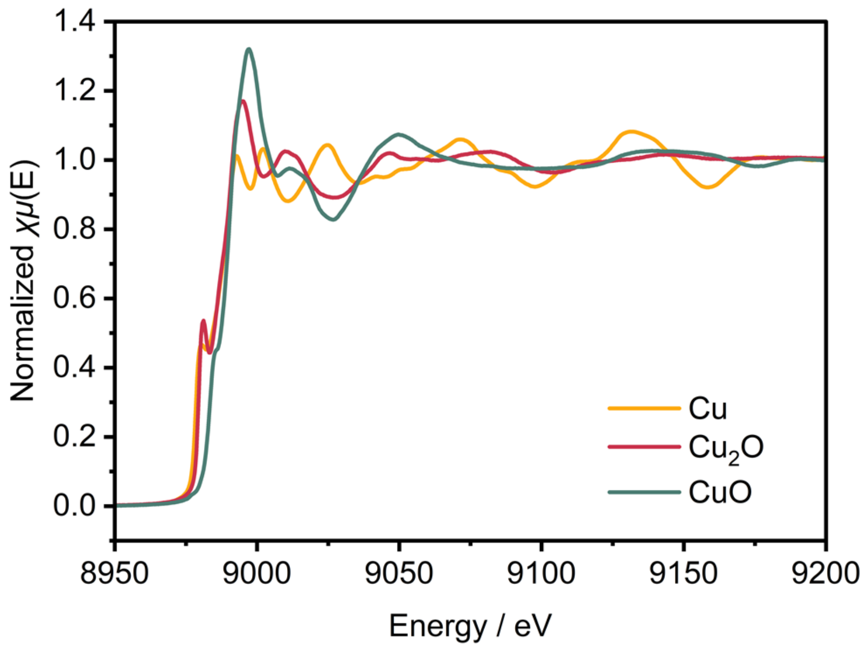

Figure S5. X-ray absorption spectra of Cu, Cu_2O, and CuO.

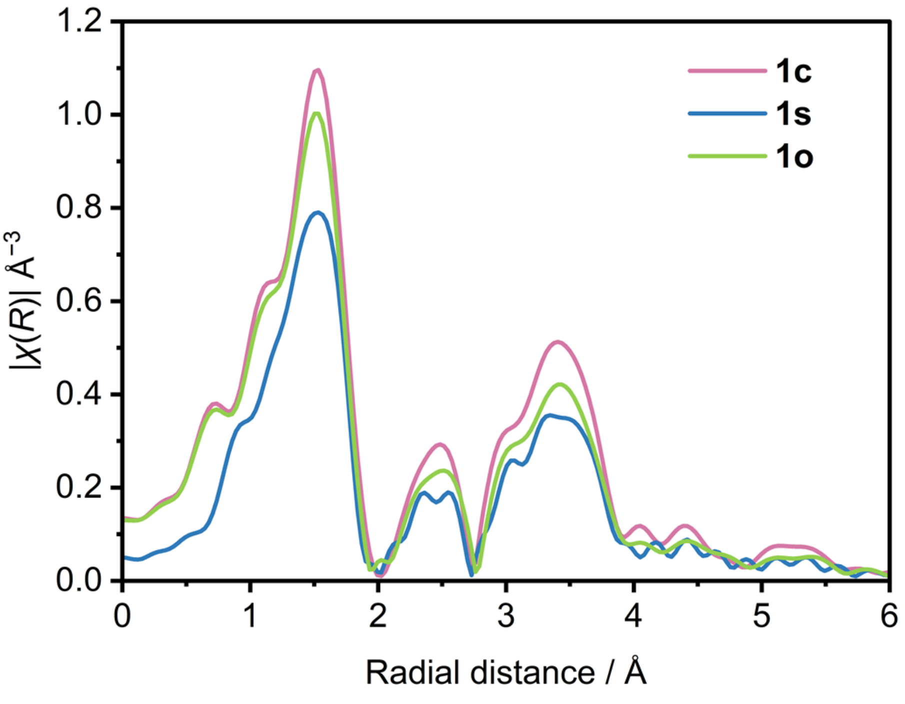

Figure S6. Fourier transform extended X-ray absorption spectra (EXAFS) of **1c**, **1s**, and **1o**.

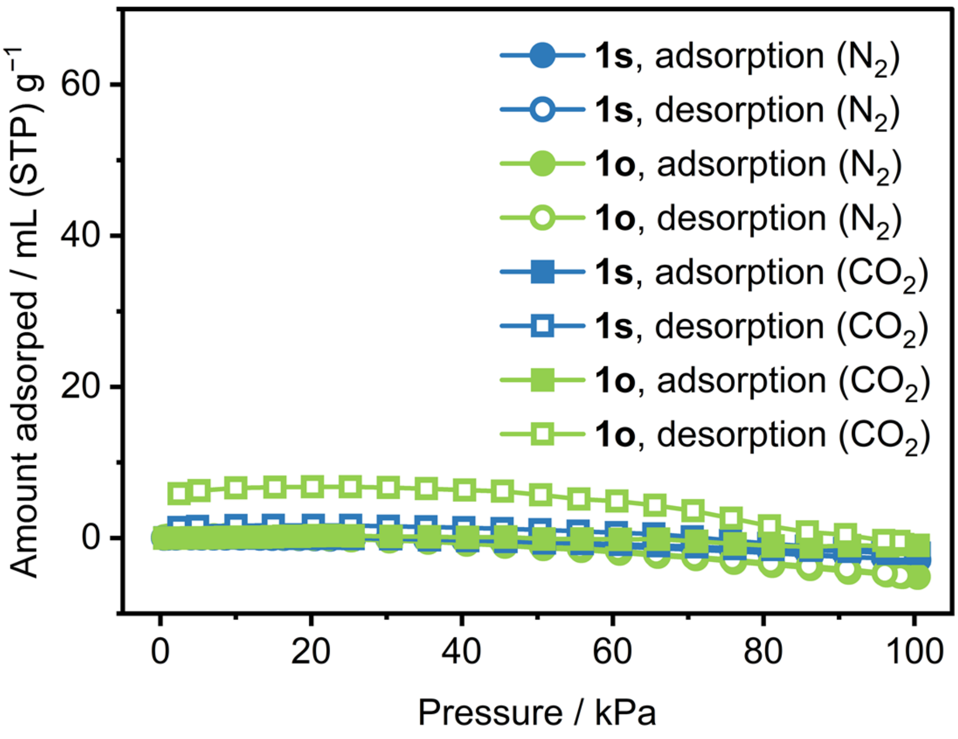

Figure S7. N_2 (77 K) and CO_2 (195 K) adsorption/desorption isotherm of **1s** and **1o**.

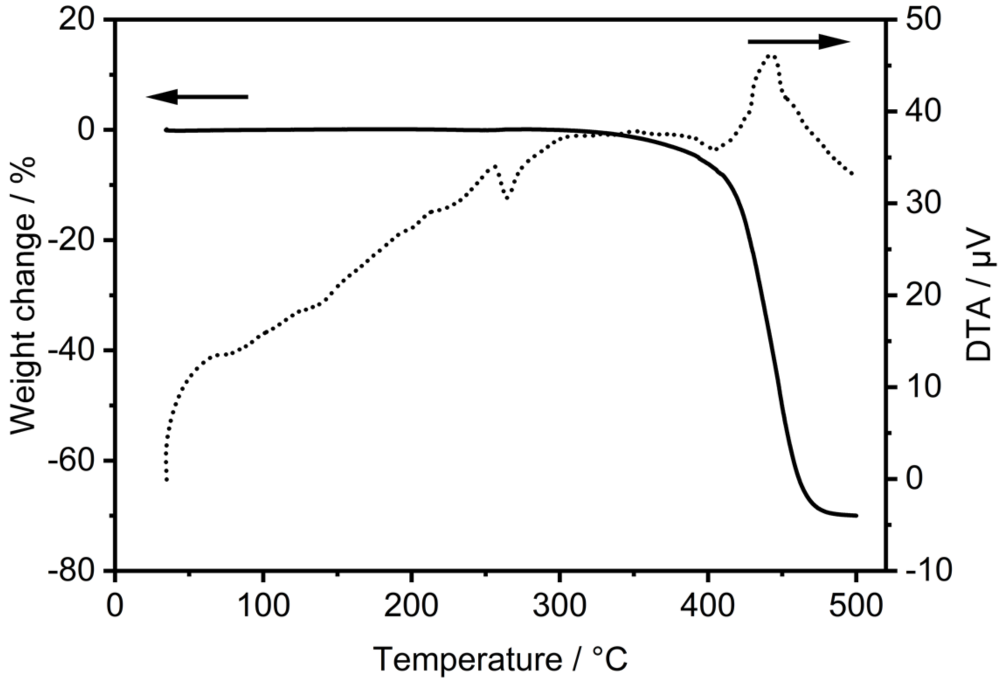

Figure S8. TG-DTA profiles of **1o** from 30 to 500 °C. Solid and dotted lines represent TGA and DTA, respectively.

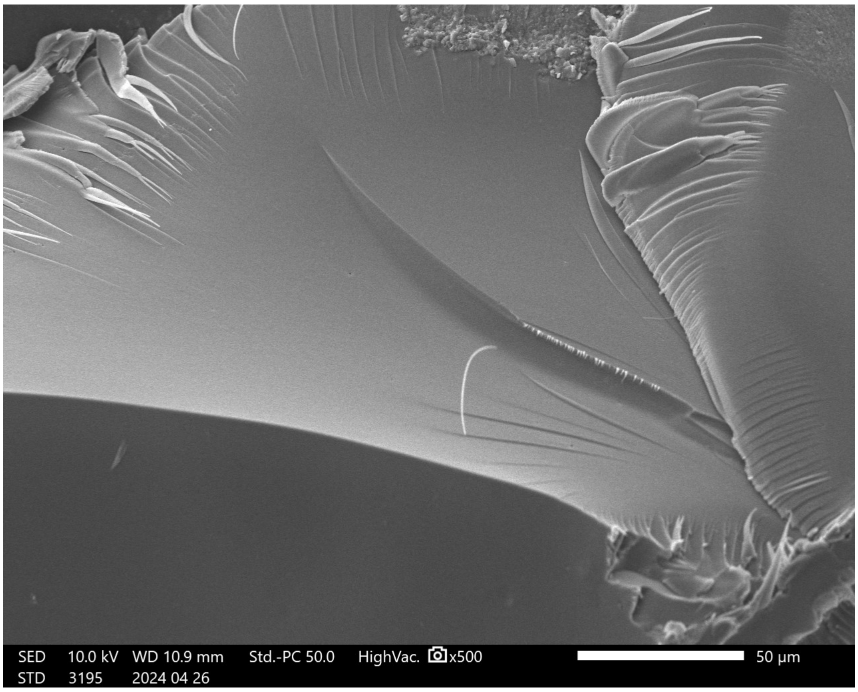

Figure S9. Scanning electron microscope (SEM) image of **1o**.

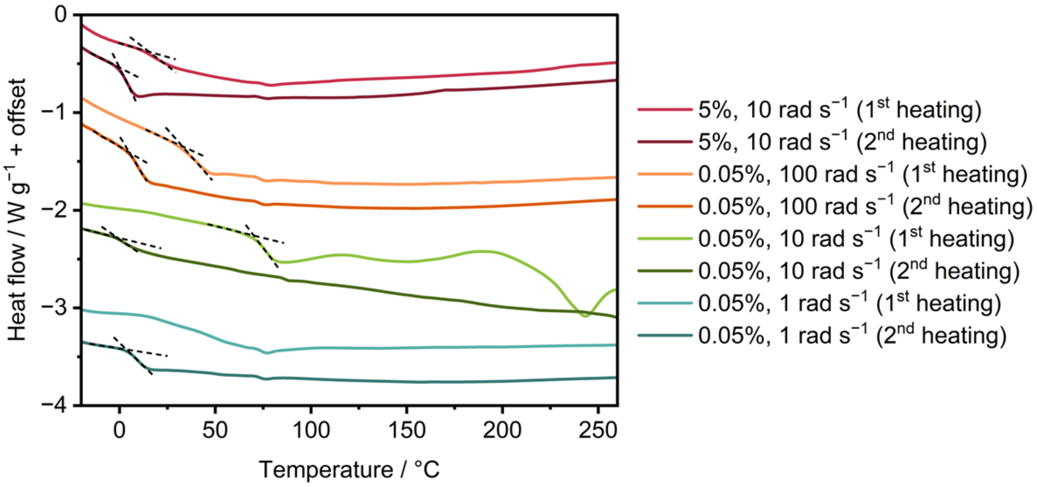

Figure S10. DSC profiles of samples prepared under different shear rates and strains. Glass transition points are indicated by hashed lines.

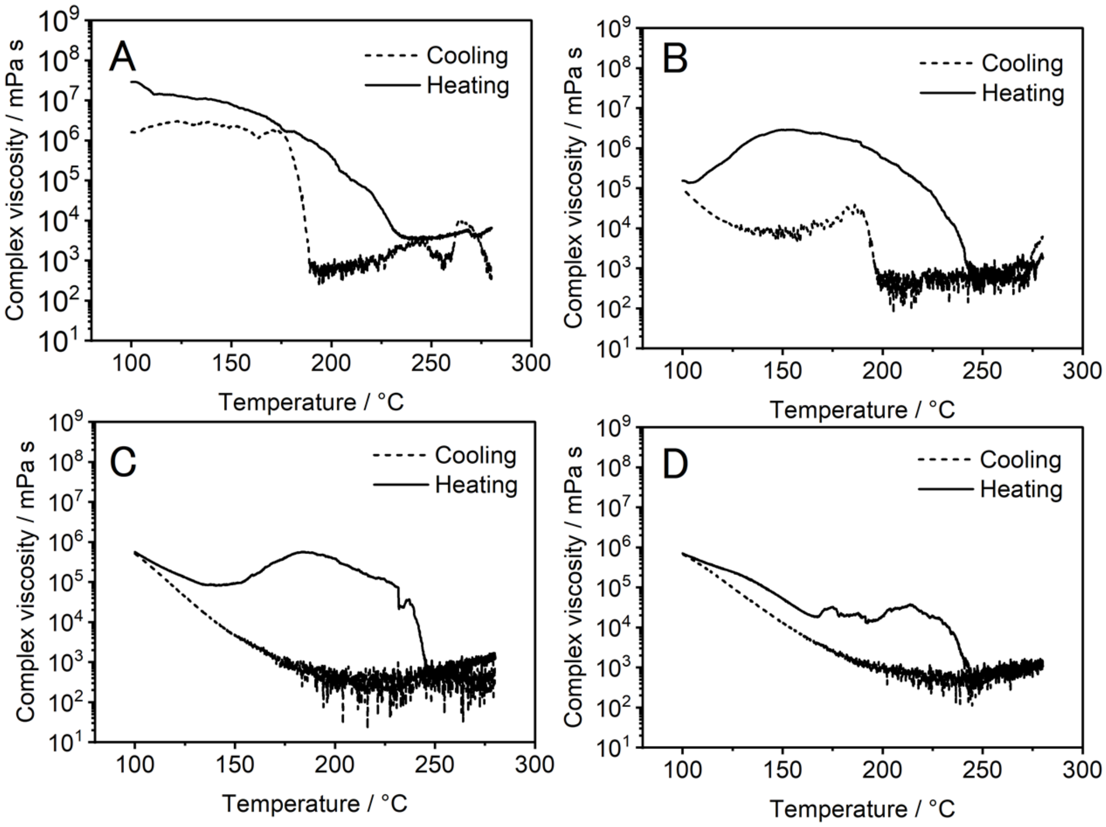

Figure S11. (A) 2nd, (B) 3rd, (C) 4th, and (D) 5th cycles of DMA of **1m** at 100–280 °C. The temperature ramping rates were 1 °C min^{-1}. Hashed lines represent cooling processes followed by each heating process indicated in solid lines. The weakening of the hysteresis should be due to the partial decomposition of the sample in each 6-hour cycle.

References

1 G. M. Sheldrick, *Acta Cryst.*, 2015, **A71**, 3–8.

2 G. M. Sheldrick, *Acta Cryst.*, 2015, **C71**, 3–8.

3 T. E. Faber and J. M. Ziman, *Philos. Mag.*, 1965, **11**, 153–173.

4 E. Lorch, *J. Phys. C: Solid State Phys.*, 1969, **2**, 229.

5 S. Kohara, M. Itou, K. Suzuya, Y. Inamura, Y. Sakurai, Y. Ohishi and M. Takata, *J. Phys. Condens. Matter*, 2007, **19**, 506101.
